# Activation and Functional Connectivity of the Left Inferior Temporal Gyrus during Visual Speech Priming in Healthy Listeners and Listeners with Schizophrenia

**DOI:** 10.3389/fnins.2017.00107

**Published:** 2017-03-15

**Authors:** Chao Wu, Yingjun Zheng, Juanhua Li, Bei Zhang, Ruikeng Li, Haibo Wu, Shenglin She, Sha Liu, Hongjun Peng, Yuping Ning, Liang Li

**Affiliations:** ^1^Beijing Key Laboratory of Behavior and Mental Health, Key Laboratory on Machine Perception, Ministry of Education, School of Psychological and Cognitive Sciences, Peking UniversityBeijing, China; ^2^School of Life Sciences, Peking UniversityBeijing, China; ^3^School of Psychology, Beijing Normal UniversityBeijing, China; ^4^The Affiliated Brain Hospital of Guangzhou Medical University (Guangzhou Huiai Hospital)Guangzhou, China; ^5^Beijing Institute for Brain Disorder, Capital Medical UniversityBeijing, China

**Keywords:** speech recognition, cocktail-party problem, lipreading, visual speech priming, informational masking, unmasking, schizophrenia, inferior temporal gyrus

## Abstract

Under a “cocktail-party” listening condition with multiple-people talking, compared to healthy people, people with schizophrenia benefit less from the use of visual-speech (lipreading) priming (VSP) cues to improve speech recognition. The neural mechanisms underlying the unmasking effect of VSP remain unknown. This study investigated the brain substrates underlying the unmasking effect of VSP in healthy listeners and the schizophrenia-induced changes in the brain substrates. Using functional magnetic resonance imaging, brain activation and functional connectivity for the contrasts of the VSP listening condition vs. the visual non-speech priming (VNSP) condition were examined in 16 healthy listeners (27.4 ± 8.6 years old, 9 females and 7 males) and 22 listeners with schizophrenia (29.0 ± 8.1 years old, 8 females and 14 males). The results showed that in healthy listeners, but not listeners with schizophrenia, the VSP-induced activation (against the VNSP condition) of the left posterior inferior temporal gyrus (pITG) was significantly correlated with the VSP-induced improvement in target-speech recognition against speech masking. Compared to healthy listeners, listeners with schizophrenia showed significantly lower VSP-induced activation of the left pITG and reduced functional connectivity of the left pITG with the bilateral Rolandic operculum, bilateral STG, and left insular. Thus, the left pITG and its functional connectivity may be the brain substrates related to the unmasking effect of VSP, assumedly through enhancing both the processing of target visual-speech signals and the inhibition of masking-speech signals. In people with schizophrenia, the reduced unmasking effect of VSP on speech recognition may be associated with a schizophrenia-related reduction of VSP-induced activation and functional connectivity of the left pITG.

## Introduction

How are people able to detect, locate, and identify ecologically important sounds in complex acoustic environments? Recognizing target speech under noisy “cocktail-party” conditions with multiple-people talking is one of the most difficult tasks where listeners need to facilitate the perceptual segregation between target speech and masking speech using some perceptual and/or cognitive cues (Freyman et al., 2004; Helfer and Freyman, 2005, 2009; Schneider et al., 2007; Wu et al., 2012, 2013a,b; Zheng et al., 2016). Lipreading is one of the cues that can help people follow the target-speech stream of the attended speaker under “cocktail-party” conditions (Helfer and Freyman, 2005; Wu et al., 2013a,b).

In a face-to-face conversation, speech information contained in speech lipreading is both redundant and complementary to speech sounds (Summerfield, 1979). More in detail, the visual-auditory temporal synchrony (temporally co-modulated visual and auditory information) indicates the distinctive rate and dynamic phase of target-speech syllables, and consequently it both facilitates listeners' selective attention to the time windows that contain target syllables and forms the expectation of the forthcoming components of the target stream (Wright and Fitzgerald, 2004). Moreover, the degree of mouth opening of the target talker is related the overall amplitude contour of speech (Summerfield, 1992; Grant and Seitz, 2000). Finally, speech-lipreading signals also contain important phonetic information, including that of vowels, diphthongs, and place of articulation of consonants (Summerfield, 1992).

In a noisy environment, when a listener feels it difficult to comprehend what a talker has said in a face-to-face conversation, the listener usually asks the talker to repeat the attended sentence(s). The beneficial effect of “say-it-again” can be caused by viewing a talker's movements of speech articulators (i.e., the unmasking effect of visual-speech prime, VSP, Wu et al., 2013a,b). The unmasking effect of VSP is normally based on the incorporation of several perceptual/cognitive processes, including the processing of speech information contained in lipreading, working memory of lipreading information, audiovisual integration during the co-presentation of the target speech and the masking speech, selective attention on target speech, and suppression of irrelevant masking signals (Wu et al., 2013a). People with schizophrenia, however, show impaired ability of using the temporally pre-presented lip-reading cue to improve target-speech identification against speech masking (Wu et al., 2013b), possibly suggesting a combined effect of working-memory deficits (Forbes et al., 2009), cross-modal-integration deficits (Ross et al., 2007; Wu et al., 2013b), and object-oriented-attention deficits (Zheng et al., 2016; Wu et al., 2016).

Over the past decade, considerable progress has been made in localizing the brain regions that are involved in either processing of speech lipreading (Ludman et al., 2000; Campbell et al., 2001; Calvert and Campbell, 2003; Capek et al., 2008; Xu et al., 2009; Bernstein and Liebenthal, 2014) or perception of masked speech (Scott et al., 2004; Badcock and Hugdahl, 2012; Scott and McGettigan, 2013). For example, compared to stilled visual speech images, moving visual speech images (lipreading) induce activation in the bilateral lingual gyrus, superior/middle temporal cortex, bilateral parietal lobule, and bilateral inferior frontal gyrus (IFG) (Calvert et al., 1997; Calvert and Campbell, 2003). Particularly, the inferior temporal gyrus (ITG) is activated by observation of face gestures (Bernstein et al., 2011), speaking faces (Ludman et al., 2000; Campbell et al., 2001), or symbolic gestures (Xu et al., 2009). As for the processing of masked speech, there is extensive, level-independent activation in the dorsolateral temporal lobes associated with the contrast of speech-in-speech over speech-in-noise conditions (Scott et al., 2004; Scott and McGettigan, 2013). Moreover, fMRI-recording studies on audiovisual integration have shown that increased BOLD signals are observed in the bilateral posterior superior temporal sulcus (pSTS) when processing audiovisual speech with degraded auditory stimulation (Szycik et al., 2008), and in the left ITG when processing multi-modal semantic signals associated with the meaning of speech (Wise et al., 1991; Vandenberghe et al., 1996; Mummery et al., 1999; Giraud and Truy, 2002). However, the neural mechanism underlying the unmasking effect of VSP on target-speech recognition against speech masking remains unknown.

Up to date, it has not been clear whether the brain substrates underlying the unmasking effect of VSP are impaired in people with schizophrenia. It has been shown that deficits of audiovisual integration are amongst the most consistent perceptive and cognitive impairments in people with schizophrenia (Surguladze et al., 2001; de Gelder et al., 2005; Foucher et al., 2007; de Jong et al., 2009; Szycik et al., 2009; Williams et al., 2010). Less activation in the right IFG, bilateral superior/middle temporal gyri, and left posterior ITG have been observed in people with schizophrenia while performing the silent lip-reading task (Surguladze et al., 2001). People with schizophrenia also show an inverted response direction in the right medial frontal gyrus, right IFG, bilateral caudate and fusiform gyrus in the congruent vs. incongruent audiovisual task (Szycik et al., 2009). In particular, people with schizophrenia exhibit deficits in benefiting from visual speech (lipreading) information when processing auditory speech (de Gelder et al., 2005; Ross et al., 2007; Wu et al., 2013b). Thus, investigation of the brain substrates underlying the unmasking effect of VSP may further our understanding of schizophrenia.

Using the functional magnetic resonance imaging (fMRI) method, this study was to investigate the brain substrates underlying the unmasking effect of VSP in healthy listeners and in listeners with schizophrenia.

## Methods

### Participants

Patients with schizophrenia were diagnosed with the Structured Clinical Interview for DSM-IV (SCID-DSM-IV) (First et al., 1997), and were recruited in the Affiliated Brain (Huiai) Hospital of Guangzhou Medical University with the recruiting criteria used previously (Wu et al., 2012; Zheng et al., 2016). Patients with diagnoses of schizoaffective or other psychotic disorders were not included. Some potential patient participants were excluded from this study if they had comorbid diagnoses, substance dependence, or other conditions that affected experimental tests (including hearing loss, a treatment of the electroconvulsive therapy (ECT) within the past 3 months, a treatment of trihexyphenidyl hydrochloride with a dose of more than 6 mg/day, or an age younger than 18 or older than 59).

Demographically matched healthy participants were recruited from the community around the hospital with the recruiting criteria used previously (Wu et al., 2012; Zheng et al., 2016). They were telephone-interviewed first and then those who passed the interview were screened with the SCID-DSM-IV as used for patient participants. None of the selected healthy participants had either a history of Axis I psychiatric disorder as defined by the SCID-DSM-IV.

Patient participants, patient guarantees, and healthy participants gave their written informed consent for participation in this study. The procedures of this study were approved by the Independent Ethics Committee (IEC) of the Affiliated Brain (Huiai) Hospital of Guangzhou Medical University.

Twenty-five patients with schizophrenia and 17 healthy listeners participated in this study. Three patients and 1 control participant were excluded from data analyses due to excessive head movement during fMRI scanning (>3 mm in translation or >3° in rotation from the first volume in any axis). The remaining 22 patients (14 males and 8 females, with age 29.0 ± 8.1 years) and 16 healthy participants (7 males and 9 females, with age 27.4 ± 8.6 years) were included in fMRI data analyses and behavioral testing. All the participants were right-handed with pure-tone hearing thresholds (< 30 dB HL) between 125 and 8,000 Hz for the two ears, and had normal or corrected-to-normal vision. Their first language was Mandarin Chinese. All patient participants received antipsychotic medications during this study with the average chlorpromazine equivalent of 521 mg/day (based on the conversion factors described by Woods, 2003) and were clinically stable during their participation. For the purpose of improving sleeping, some of the patient participants also received benzodiazepines based on doctors' advice. The locally validated version of the Positive and Negative Syndrome Scale (PANSS) tests (Si et al., 2004) was conducted on the day of fMRI scanning for all participants. The characteristics of patient participants and healthy participants are shown in Table 1.

**Table 1:** **Characteristics of patients with schizophrenia and healthy controls**.

**Basic characteristic**	**Patients with schizophrenia (*n* = 22)**	**Healthy people (*n* = 16)**
Age (years ± SD)	29.00 (8.05)	27.44 (8.63)
Male % (n)	63.55 (14)	43.75 (7)
Education (years ± SD)	13.14 (2.47)	15.00 (2.58)
MID (years ± SD)	5.49 (4.05)	NA
PANSS total	53.64 (6.04)	NA
PANSS positive	14.86 (4.16)	NA
PANSS negative	11.45 (4.03)	NA
PANSS general	34.56 (9.62)	NA
Medication	Patient Number	
Typical	5	NA
Atypical	19	NA
Typical and atypical^a^	2	NA
Chlorpromazine equivalent	Mean (SD): 521 (223)	NA
	Range: 225–1,000	

*MID, mean illness duration; NA, not applicable; PANSS, Positive and Negative Syndrome Scale; SD, standard deviation*.

a *Two patients received both typical and atypical antipsychotic medications*.

### Procedures of the fMRI experiment

#### Stimuli and design

**Figure 1:**
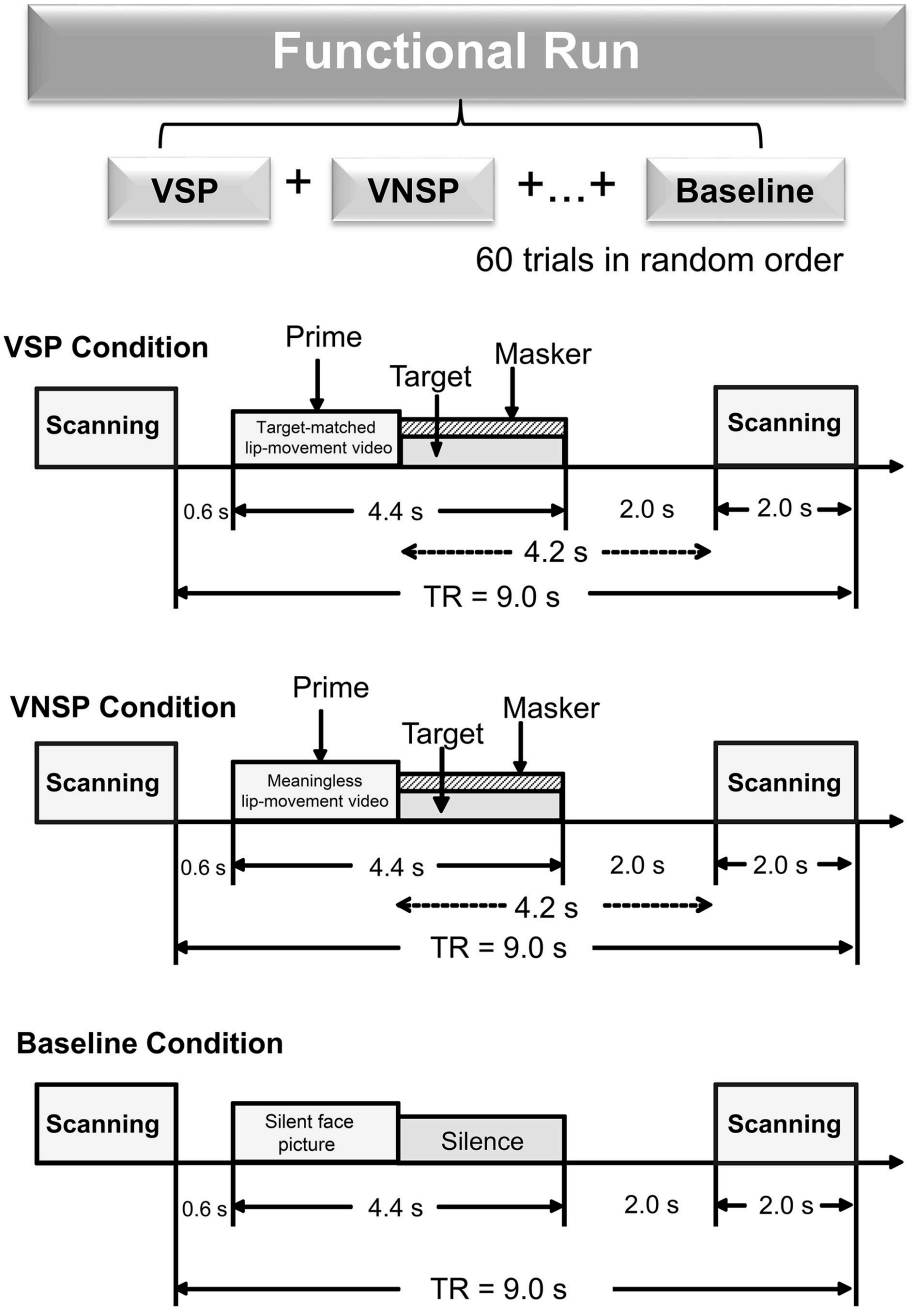
**A functional run comprised 60 trials (20 trials for each of the three conditions: VSP, VNSP and baseline simulation) presented in random order**. Sparse temporal sampling was used to acquire images. Trial structures of each of the three conditions were illustrated respectively. Under the visual priming condition, a priming stimulus was presented 600 ms after the scanning, then the target and masker sounds were co-presented, and terminated at the same time. The midpoint of the stimulus was presented 4.2 s prior to the next scanning. VSP, visual speech priming; VNSP, visual non-speech priming; TR, time to repeat.

There were three types of stimuli: (auditory) target-speech stimuli, (auditory) masking-speech stimuli, and visual priming stimuli. The target-speech stimuli used in both the fMRI experiment and the behavioral testing were “nonsense” Chinese phrases with 3 words and each word contained 2 syllables (in total 6 syllables in a phrase). These phrase were syntactically ordinary but not semantically meaningful (Yang et al., 2007; see Wu et al., 2013b), and spoken by a young female talker (Talker A). For example, the English translation of a phrase is “retire his ocean” (keywords are underlined). Obviously, the phrase frame provided no contextual support for recognizing individual keywords. The duration of a target phrase was around 2,200 ms (Figure 1).

The masking-speech stimuli were a 47-s loop of digitally-combined continuous recordings for Chinese nonsense sentences (whose keywords did not appear in target sentences) spoken by two different young female talkers (Yang et al., 2007). In a trial, the masker started at a random position of the loop and the duration of the masker was adjusted equal to that of the target phrase.

There were two types of visual priming stimuli: (1) (lipreading) visual-speech priming (VSP) stimuli, whose associated content and duration were identical to those of their corresponding target phrases; (2) (lipreading) visual non-speech priming (VNSP) stimuli, whose facial movements were not related to any speech content (i.e., alternations of mouth-open and mouth-close movements, used as the control stimulation condition for the VSP-stimulation condition; Calvert et al., 1997; Calvert and Campbell, 2003) and whose durations were also identical to those of the following target phrases. To minimize the facial identity effect, only the lower half part of the priming talker's face was displayed (Wu et al., 2013a,b). The duration of visual priming stimuli were identical to that of target speech stimuli.

As the general controlling condition for both the VSP condition and the VNSP condition (to control both the facial features and the auditory non-speech features for VSP and VNSP conditions), a baseline-stimulation condition was introduced with presenting both a still face (duration = 2,200 ms) and a period of silence (duration = 2,200 ms) (Figure 1). The same talker's face was used for both the 2 priming conditions and the baseline-stimulation condition.

The whole-course scanning consisted of a 10-min visual-priming functional run and an 8-min structure-scanning run. An event-related fMRI design was used for the functional run. In total, there were 60 scanning trials for the functional run (20 trials for each of the 3 conditions: VSP, VNSP, and baseline stimulation). For an individual participant, the 60 trials across the 3 conditions were presented with a random order.

The sparse-temporal imaging strategy was used to avoid the effect of machine scanning noise: the acoustic stimulus presentation was temporally positioned so that the stimulus midpoint was 4,200 ms before the onset of the next scanning. Thus, the stimulus-evoked hemodynamic responses peaked during the scanning period (Wild et al., 2012; Zheng et al., 2016).

The sound pressure level of target speech was 60 dB SPL (after attenuation by earplugs) in the fMRI experiment, and the signal-to-masker ratio (SMR) was set as −4 dB.

In a scanning trial under either the VSP or VNSP condition (Figure 1), the priming stimulus was presented 600 ms after the offset of the last scanning trial. Immediately after the prime presentation, the target and masker were presented and terminated simultaneously. To maintain participants' attention to the stimuli, at the end of a trial (after the acoustic stimuli were presented), participants were instructed to use button pressing with their right index finger to indicate whether the pre-presented lipreading prime was matched to the target phrase or not. Scores of button-pressing was recorded. A brief training was provided to ensure that participants understood the instructions and knew how to conduct their button-pressing responses. Before the fMRI experiment and the behavioral testing, participants also received a specific training to distinguish VSP stimuli from VNSP stimuli. Speech stimuli used in training were different from those used in formal experiments.

#### Equipment

During fMRI scanning, acoustic stimuli were presented through a magnetic resonance-compatible pneumatic headphone system (SAMRTEC, Guangzhou, China) driven by Presentation software (Version 0.70). Visual stimuli were presented through a liquid-crystal-display screen positioned on the head-coil (SAMRTEC, Guangzhou, China). A 3.0-Tesla Philips Achieva MRI scanner (Veenpluis 4-6,5680 DA Best, Netherlands) was used to acquire blood oxygenation level dependent (BOLD) gradient echo-planar images (spatial resolution: 64 × 64 × 33 matrix with 3.44 × 3.44 × 4.6 mm^3^; acquisition time: 2,000 ms; time to repeat: 9,000 ms; echo time: 30 ms; flip angle: 90°; field of view: 211 × 211 mm). It provided high-resolution T1-weighted structural images (256 × 256 × 188 matrix with a spatial resolution of 1 × 1 × 1 mm^3^, repetition time: 8.2 ms; echo time: 3.8 ms; flip angle: 7°).

### fMRI data processing and analyses

#### Pre-processing

All fMRI data were processed and analyzed using the Statistical Parametric Mapping (SPM8, the Wellcome Trust Centre for Neuroimaging, London, UK). The pre-processing of data included the following stages: (1) The functional images were corrected for head movements. (2) The anatomical images were co-registered with the mean realigned images and were normalized to standard template (ICBM space) using the SPM8 unified segmentation routine. (3) All functional images were warped using deformation parameters generated from the normalization process, including re-sampling to a voxel size of 3.0 × 3.0 × 4.0 mm^3^. (4) Spatial smoothing was conducted using a Gaussian kernel with 8 mm full-width at half maximum (FWHM). Due to the long TR of this sparse-imaging paradigm, no slice timing correction was necessary.

#### Whole brain analyses

Random effect analyses contained two processing levels. At the first level, the onsets and durations for the functional run were modeled using a General Linear Model (GLM) according to the condition types. Three conditions (VSP, VNSP, and the baseline) were included in the model. Time series on the six realignment parameters of head movement were also included as regressors of no interest in the GLM design matrix to account for residual movement-related effects (Friston et al., 1996). Contrasts of “VSP > baseline,” “VNSP > baseline” and “VSP > VNSP” were made for each participant at the first level. At the second level, random-effect analyses were conducted based on the statistical parameter maps from each individual participant to allow population inference. Contrast images of “VSP > baseline,” “VNSP > baseline” from the first-level analysis in each participant were entered into the second-level full-factor 2 (group: control, patient) by 2 (condition: “VSP > baseline,” “VNSP > baseline”) ANOVA to detect interaction between group and priming type. Contrast images of “VSP > VNSP” from the first-level analysis in each participant were entered into a second-level two-sample *t*-test to explore the group differences in brain activation induced by VSP directly. For the whole-brain analyses, peak signals that were statistically significant at *p*-value less than 0.05 [False Discovery Rate (FDR) corrected] were reported.

#### Region-of-interest (ROI) analyses

As mentioned above, the contrast of “VSP > VNSP” was computed to map the brain regions that were activated by the processing of the speech lipreading-induced priming (the VSP). These brain regions were called VSP-activated brain regions.

ROI analyses and correlation analyses were conducted to identify the VSP-activated brain regions that were also correlated to the “VSP effect” of speech recognition in the behavioral testing (the difference in percent correct of target-speech recognition between the VSP condition and the VNSP condition). More in detail, first, based on the group mean “VSP > VNSP” contrast (*p* < 0.05, FDR corrected), a functionally-defined ROI was a sphere with a radius of 5 mm centered at MNI coordinates of peak activation. In addition, the parameter estimates of signal intensity of each ROI under each condition were extracted from each individual participant (MarsBaR: region of interest toolbox for SPM; http://marsbar.sourceforge.net/). Moreover, for each ROI, the contrast value (CV) for the speech-lipreading priming process (i.e., the parameter estimate difference between the VSP condition and the VNSP condition) was calculated (Wild et al., 2012). Finally, partial correlation analyses (age, hearing threshold, education level, and sex were the covariates) were conducted using SPSS 16.0 software to investigate the correlation between the brain activation induced by the “VSP > VNSP” contrast in the fMRI measuring and the unmasking effect of the VSP stimulus in the behavioral testing.

#### Functional connectivity analyses: psychophysiological interaction

Psychophysiological interaction analyses (Friston et al., 1997) were conducted to identify brain regions that showed significantly increased or reduced covariation (i.e., functional connectivity) with the seed region activity related to the VSP (VSP > VNSP) effect in both healthy participants and participants with schizophrenia.

For both healthy participants and participants with schizophrenia, the seed ROI was defined in the brain region that exhibited more activation in healthy participants than that in people with schizophrenia from the whole brain ANOVA analyses. The seed ROI in each individual participant was defined as a sphere with 5-mm-radius centered at the peak MNI coordinate in the seed region. First, the time series of seed region were extracted, and the PPI regressors which reflected the interaction between psychological variable (VSP vs. VNSP) and the activation time course of the seed ROI were calculated. Second, the individual contrast images (regressors) were subsequently subjected to the second-level one-sample *t*-tests in each of the participant groups to identify the brain regions showing increased co-variation with the activity of the seed region in analyses of the VSP condition vs. the VNSP condition. Finally, contrast images of each participant in the control group and patient group were entered into the second-level two-sample *t*-tests for group comparisons. In PPI analyses, peak signals that were statistically significant at *p*-value less than 0.05 (FDR corrected) were reported.

### Behavioral testing

The behavioral testing was conducted after the fMRI scanning experiment. Acoustic signals, as used in the fMRI experiment, were calibrated by a sound-level meter (AUDit and System 824, Larson Davis, USA) and delivered from a notebook-computer sound card (ATI SB450 AC97) to participants via headphones (Model HDA 600). The target-speech level was 60 dB SPL and the SMR was either −4 or −8 dB. There were two within-subject variables: (1) priming type (VSP, VNSP), and (2) SMR (−8, −4 dB). For each participant, there were 4 testing conditions and 20 trials (also 20 target-sentence presentations) for each condition. The presentation order for the 4 conditions (i.e., the 4 combinations of priming type and SMR) were partially counterbalanced across participants using a Latin square order.

In a trial, the participant (who was seated at the center of a quiet room) pressed the “Enter” key on a computer keyboard to start the presentation of the visual priming stimulus. Immediately after the presentation of the visual priming stimulus, the target phrase was co-presented with the masking speech (the target and masker began and terminated at the same time). After the masker/target co-presentation, the participant was instructed to loudly repeat the whole target phrase as best as he/she could. The experimenters, who sat quietly behind the participant, scored whether each of the two syllables of the keywords in the target phrase had been identified correctly. In the behavioral testing, the unmasking effect of VSP was defined as the difference in percent correct of target speech recognition between the VSP-listening condition and the VNSP-listening condition averaged across SMRs. Analyses of variance (ANOVA) were performed using SPSS 16.0 software. The null hypothesis was rejected at the level of 0.05.

## Results

### The unmasking effect of VSP on speech-recognition performance

**Figure 2:**
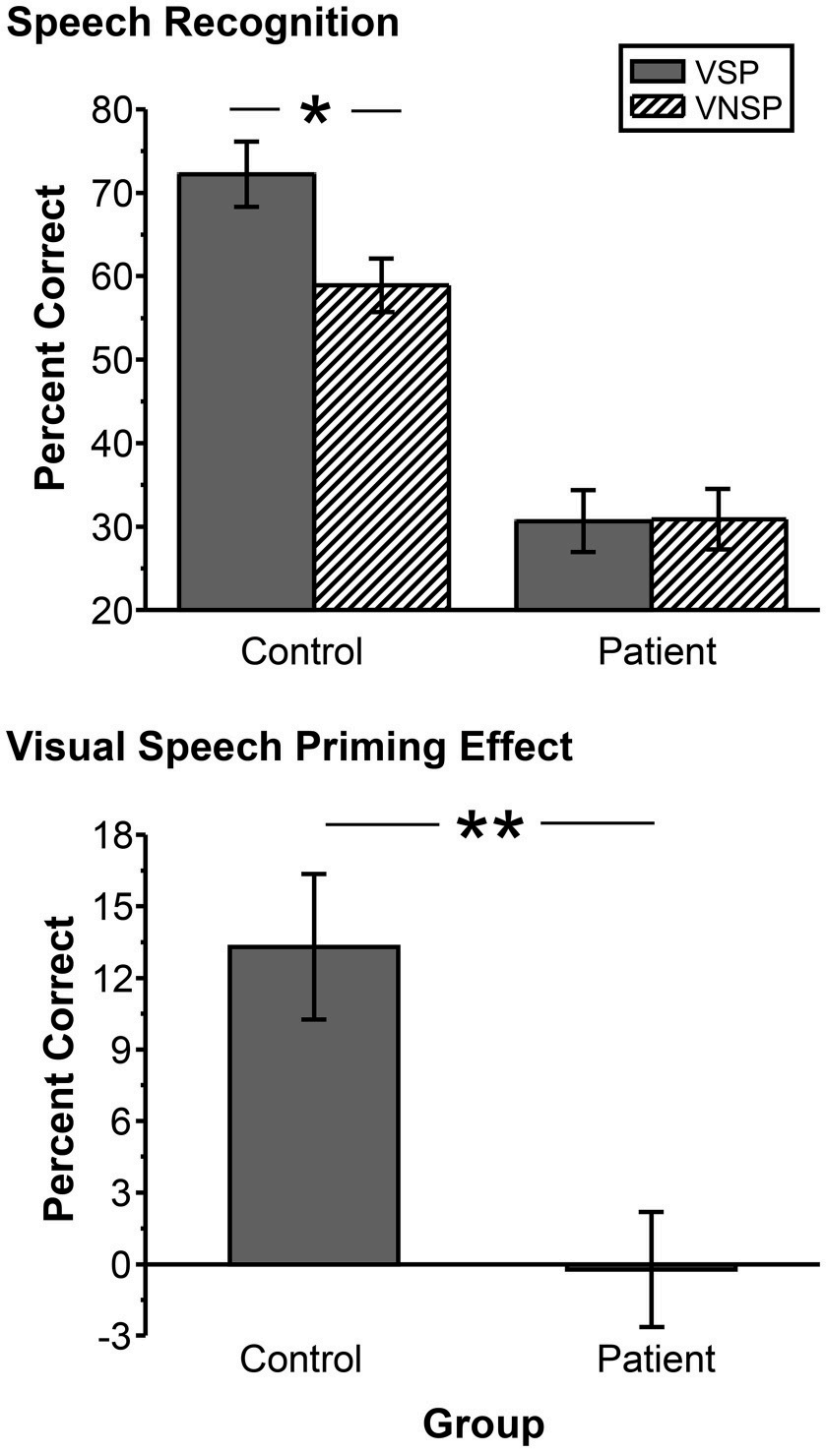
**Upper panel:** Comparisons in group-mean percent-correct recognition of the two target keywords against speech masking between the control group and the patient group under the VSP listening condition and the VNSP listening condition (averaged across SMRs of −4 and −8 dB). **Lower panel:** Difference in the group-mean unmasking effect (VSP effect: difference in percent correct of target speech recognition between VSP and VNSP conditions) between healthy participants and people with schizophrenia (averaged across SMRs of −4 and −8 dB). Error bars indicate the standard errors of the mean. VSP, visual speech priming; VNSP, visual non-speech priming. ^*^p < 0.05, ^**^p < 0.01.

Figure 2 (upper panel) shows comparisons in group-mean percent-correct recognition of the two target keywords between the healthy participants and participants with schizophrenia under the VSP condition and the VNSP condition, respectively. The 2 (group: control, patient) by 2 (priming type: VSP, VNSP) ANOVA showed that the main effect of group was significant [*F*_(1, 72)_ = 90.302, *p* < 0.001, η^2^ = 0.559], the main effect of prime was significant [*F*_(1, 72)_ = 4.548, *p* = 0.036, η^2^ = 0.059], and the interaction between group and priming type was significant [*F*_(1, 72)_ = 4.817, *p* = 0.031, η^2^ = 0.063]. Obviously, healthy participants had better speech-recognition performance than patient participants.

The control group, but not the patient group, was able to use the lipreading cue to improve target-speech recognition (*p* = 0.002 for the control group, and *p* = 0.965 for the patient group). Figure 2 (lower panel) and Figure S2 show that the VSP effect (difference in percent correct of target speech recognition between the VSP condition and the VNSP condition) was significantly higher in healthy participants than that in participants with schizophrenia (*t* = 3.519, *p* = 0.001; Cohen's *d* = 1.13; 95% CI: 0.43–1.84). Figure S1 also shows the significantly lower percent correct of button-pressing response for patients than that for healthy participants during fMRI scanning (*t* = 5.507; *p* < 0.001).

### Brain substrates associated with the unmasking effect of VSP in healthy participants

#### Brain regions activated by the VSP > VNSP contrast

**Figure 3:**
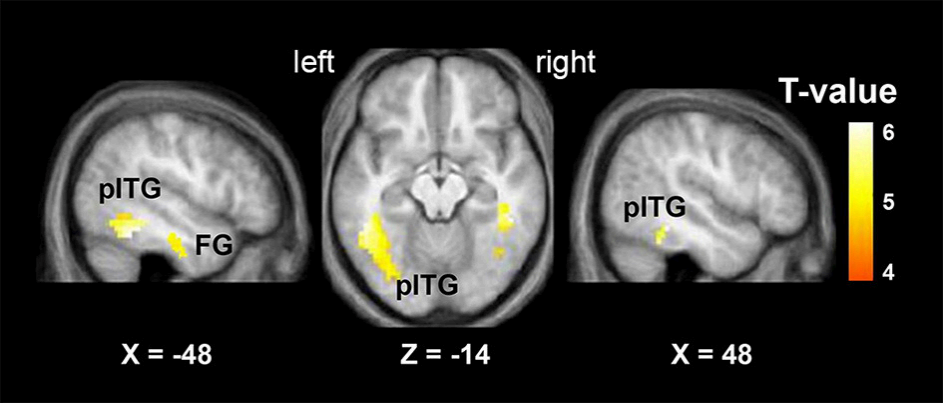
**Brain regions activated by the contrast of “VSP > VNSP” (in healthy people), including the bilateral pITG and bilateral FG**. The activation maps were thresholded at *p* < 0.05 (voxel-wise FDR corrected, *T* > 4.87) and overlaid on the group-average structural image. FG, fusiform gyrus; pITG, posterior inferior temporal gyrus; VSP, visual speech priming; VNSP, visual non-speech priming.

The “VSP > VNSP” BOLD contrast was used to determine the brain regions that were activated by the VSP-stimulation condition. The results showed that in healthy participants, but not participants with schizophrenia, compared to the VNSP condition, introducing the VSP condition significantly enhanced BOLD signals in the bilateral posterior inferior temporal gyrus (pITG) and bilateral fusiform gyrus (FG) (*p* < 0.05, voxel-wise FDR corrected) (Figure 3, Table 2). These 4 brain regions are called “VSP-activated brain regions”.

**Table 2:** **MNI coordinates of the brain regions associated with the contrast of the visual speech priming (VSP) condition against the visual non-speech priming (VNSP) condition in healthy participants**.

**Coordinates**			**Statistics**				**Location**
***X***	***Y***	***Z***	***k***	***T***	**Z-score**	***P*_FDR−corr_**	
−48	−43	−14	66	5.73	4.70	0.012	L pITG
48	−40	−14	51	5.61	4.62	0.012	R pITG
45	−43	−22	58	5.57	4.60	0.012	R Fusiform
−33	−13	−26	82	4.87	4.16	0.014	L Fusiform

*Peaks were significant at p < 0.05 (voxel-wise FDR corrected with an extent threshold of more than 40 voxels; T > 4.87) are shown in the table. MNI coordinates, k (number of voxels), T-value, and Z-scores and corrected P-values are provided. pITG, posterior inferior temporal gyrus; L, left; R, right*.

#### VSP-activated brain regions that were specifically correlated to the unmasking effect of VSP

To further search for the VSP-activated brain regions that were specifically associated with the (behavioral) unmasking effect of VSP in the behavioral testing (so-called “unmasking-correlated brain regions”), the parameter estimates of signal intensity of each of the four VSP-activated brain ROIs (i.e., left pITG, right pITG, left FG, and right FG), which was defined by a sphere with a radius of 5 mm centered at peak MNI coordinates based on the “VSP > VNSP” contrast (see Figure 3 and Table 2), were extracted and the contrast value (CV) for the “VSP > VNSP” contrast was calculated for each individual participant (Wild et al., 2012). Then, the correlation between the VSP-induced (behavioral) improvement of target-speech recognition (with age, sex, hearing threshold, and educational level controlled) and each CV (for each of the 4 ROIs) was examined.

**Figure 4:**
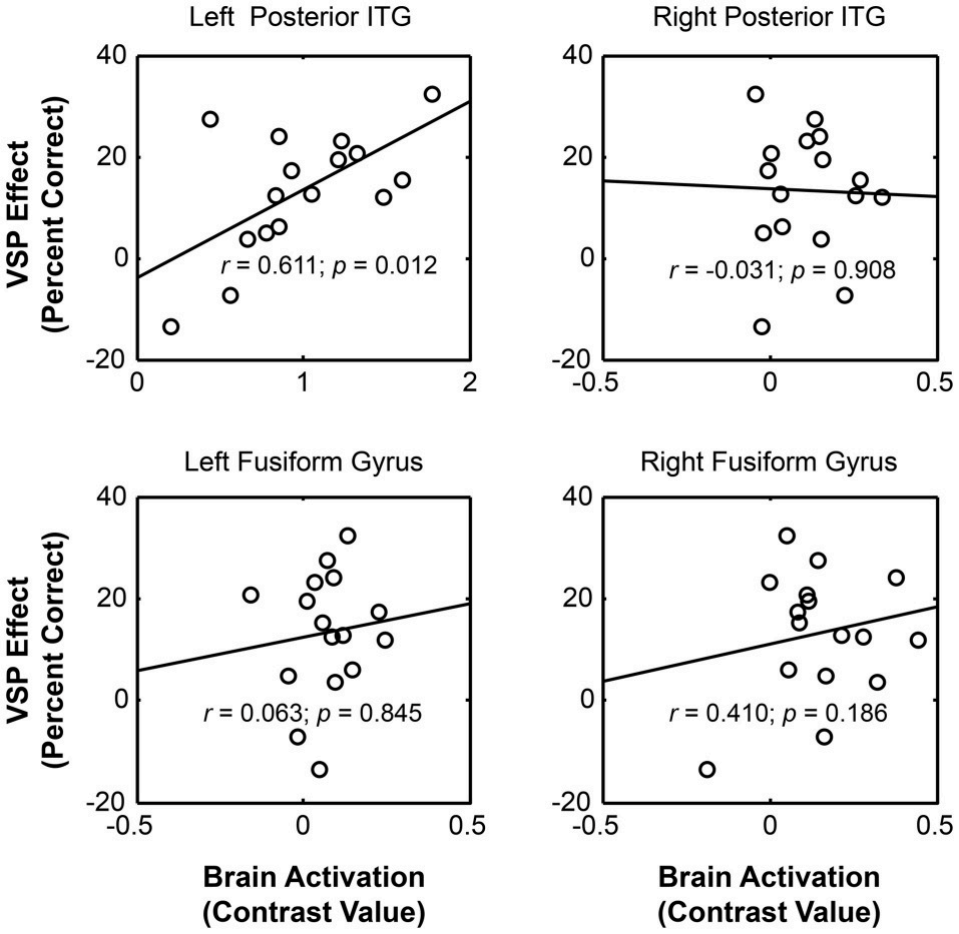
**For healthy people, significant correlation occurred between the unmasking effect of VSP (difference in percent correct of target speech recognition between the VSP condition and the VNSP condition averaged across SMRs) and the intensity of VSP-induced brain regional activity (the contrast value of VSP > VNSP) in left pITG (with age, hearing threshold, education level and sex controlled), but not that in the right ITG, left FG, or right FG**. FG, fusiform gyrus; pITG, posterior inferior temporal gyrus; VSP, visual speech priming; VNSP, visual non-speech priming.

The results showed that significant correlation occurred only between the VSP-induced CV of the left pITG and the VSP-induced improvement of target-speech recognition (*r* = 0.611, *p* = 0.012) (Figure 4). Thus, the left pITG was recognized as the brain region specifically related to the VSP-unmasking effect (i.e., the unmasking-correlated brain region).

### Differences in BOLD signals induced by VSP between healthy participants and participants with schizophrenia

The whole brain ANOVA analyses revealed no interaction between group and the priming type. The main effect of priming type was not significant [at the *p* < 0.001 (uncorrected)]. The contrast assessing the main effect of group (patient vs. control) revealed the significantly reduced activation in the bilateral triangularis of inferior frontal gyrus (TriIFG), left postcentral, left superior temporal sulcus (STS), left caudate, left fusiform, left pITG, right precentral, and right thalamus (see Figure S3, and Table S1) (F-contrasts are significant at *p* < 0.05 with voxel-wise FDR correction).

**Figure 5:**
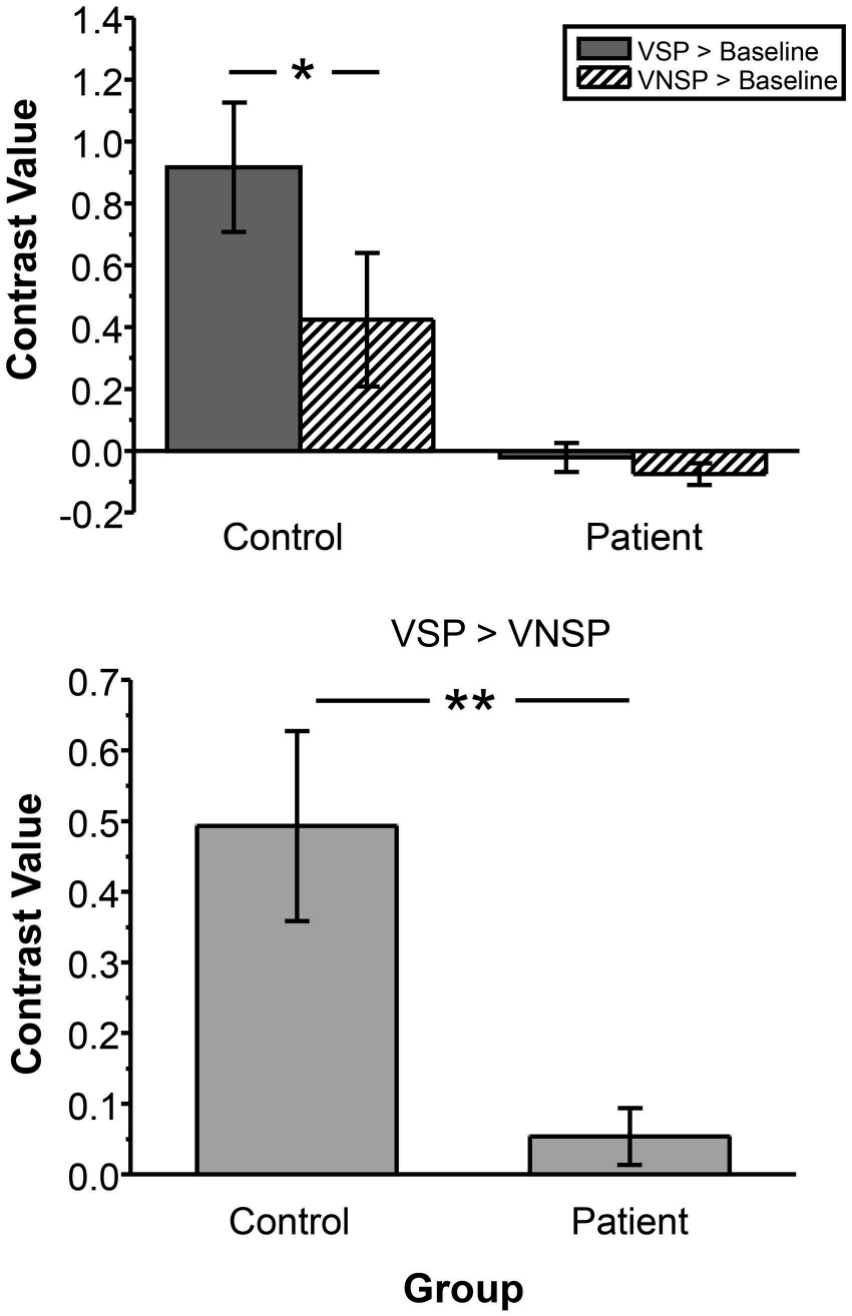
**Compared to healthy control group, patient group exhibited lower BOLD signal (contrast value) in the left pITG under either the VSP listening condition (VSP > Baseline) or the VNSP listening condition (VNSP > Baseline) (upper panel)**. Compared to healthy control group, People with schizophrenia showed lower group-mean BOLD signal in the left pITG induced by VSP (contrast value of VSP > VNSP) (lower panel). VSP, visual speech priming; VNSP, visual non-speech priming. ^*^p < 0.05, ^****^p < 0.01.

To test the difference in VSP-induced percent BOLD signal change in left pITG (which was discovered as an unmasking correlated brain region) between participants with schizophrenia and healthy participants more directly, the contrast value of VSP > Baseline, VNSP > Baseline were calculated and compared between the two participant groups. The ANOVA showed that the main effect of the group [*F*_(1, 72)_ = 29.669, *p* < 0.001, η^2^ = 0.29] and the priming type [*F*_(1, 72)_ = 4.289, *p* = 0.042, η^2^ = 0.056] on BOLD signal in left pITG were significant, and the interaction was not significant [*F*_(1, 72)_ = 2.768, *p* = 0.100] (Figure 5, upper panel). The VSP-induced contrast value (VSP > VNSP) was significantly lower in the patient group than that in the control group [*F*_(1, 36)_ = 12.649, *p* = 0.001; Cohen's *d* = 1.51; 95%CI: 0.75–2.26] (Figure 5, lower panel).

In this study, we also computed the frame-wise displacement (FD) for each time point in each participant and tested the group difference in FD between controls and people with schizophrenia. Motion-related artifact might impact findings for group difference (Yan et al., 2013), even though task-fMRI is much more tolerant to head motion than rest-fMRI (Friston et al., 1996), particularly when the presence of motion-related noise or the motion self is unrelated to the task. We did not find significant difference in mean FD at each time point between controls and patients (see Figure S4). Moreover, for the group comparison, we have computed the data with individual mean FD included as a regressor of no interest. The group difference with individual mean FD regressed out were very similar to those without individual mean FD regressed out. Thus, in this study we reported the results without the mean FD regressed out.

### Functional connectivity of the left pITG associated with the reduced VSP effect in participants with schizophrenia

A psychophysiological interaction analysis was conducted to examine the differential functional connectivity of the left pITG under the VSP condition compared to the VNSP condition between healthy participants and participants with schizophrenia. For both healthy participants and participants with schizophrenia, the seed ROI of left pITG was defined as the brain region which exhibited more activation in healthy participants than patients, with the coordinate of the peak activation of [−36, −52, −18].

**Figure 6:**
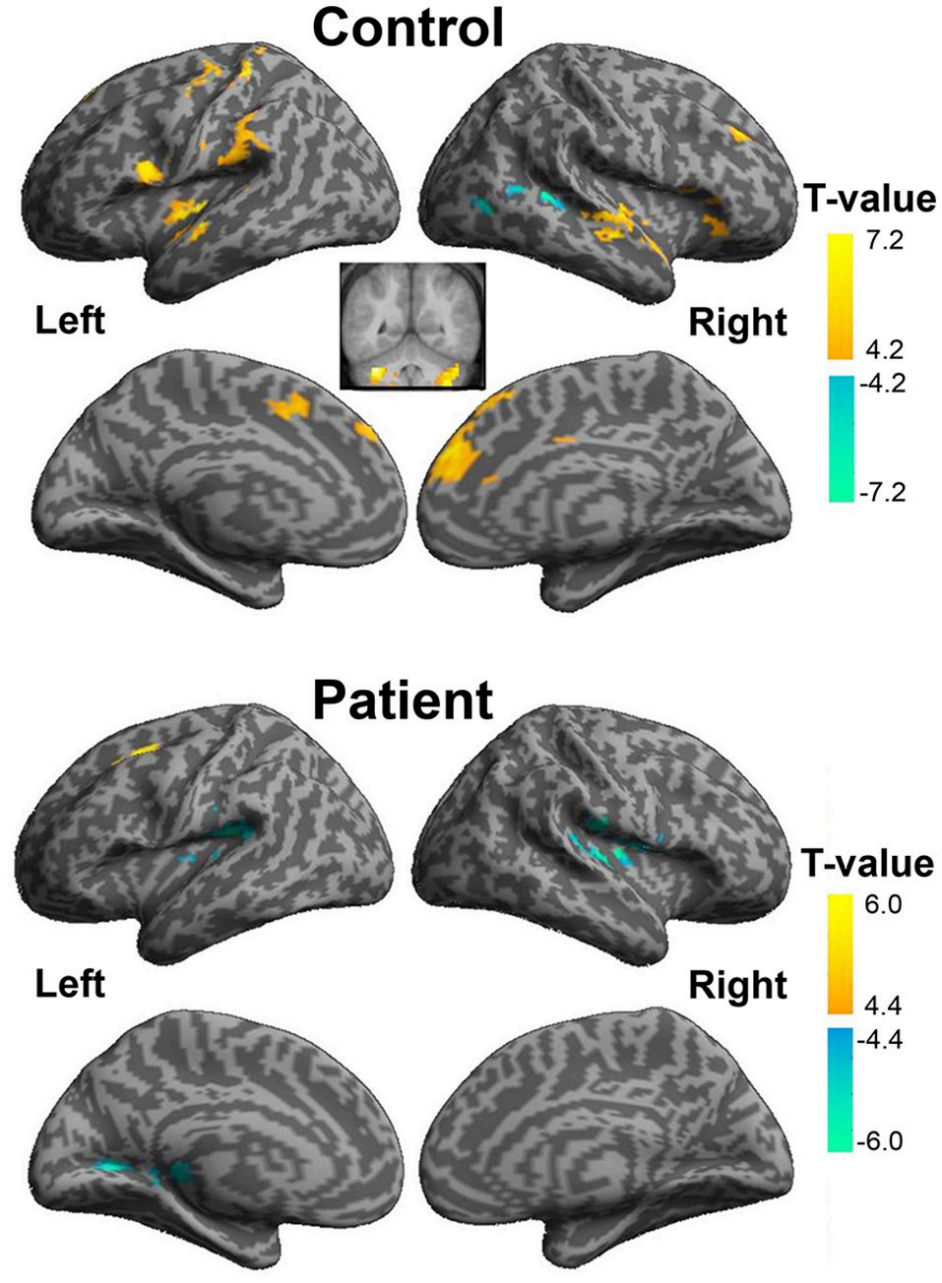
**Psychophysiological interaction analyses of the visual-speech priming effect (VSP > VNSP) for healthy participants (upper panel) and people with schizophrenia (lower panel)**. The areas in hot color-map indicate the enhanced functional connectivity with the left pITG during “VSP > VNSP,” and the regions in cool color-map indicate the reduced functional connectivity with the left pITG during “VSP > VNSP.” The activation maps were thresholded at *p* < 0.05 (voxel-wise FDR corrected, *T* > 4.88) and displayed on a template brain surface of inflated cortex from SPM8. VSP, visual speech priming; VNSP, visual non-speech priming.

In healthy participants, enhanced functional connectivity with the left pITG for the “VSP vs. VNSP” contrast occurred in the bilateral STG, bilateral medial superior frontal gyrus (mSFG), bilateral cerebellum, left precentral cortex, left postcentral cortex, left opercularis IFG, left insular, left SupraMarginal and left supplementary motor area (SMA), and right middle frontal gyrus (MFG). On the other hand, reduced functional connectivity was observed in the right posterior STG and right middle occipital cortex (Figure 6 upper panel and Table S2).

In participants with schizophrenia, enhanced functional connectivity with the left pITG for the “VSP vs. VNSP” contrast was observed in the left SFG. Reduced functional connectivity was observed in the bilateral rolandic operculum, left fusiform, left lingual, right supra-marginal area, and right thalamus. Thus, participants with schizophrenia showed a different whole brain pattern of functional connectivity with left pITG for the contrast of the VSP condition vs. the VNSP condition (Figure 6 lower panel, also see Table S2).

**Figure 7:**
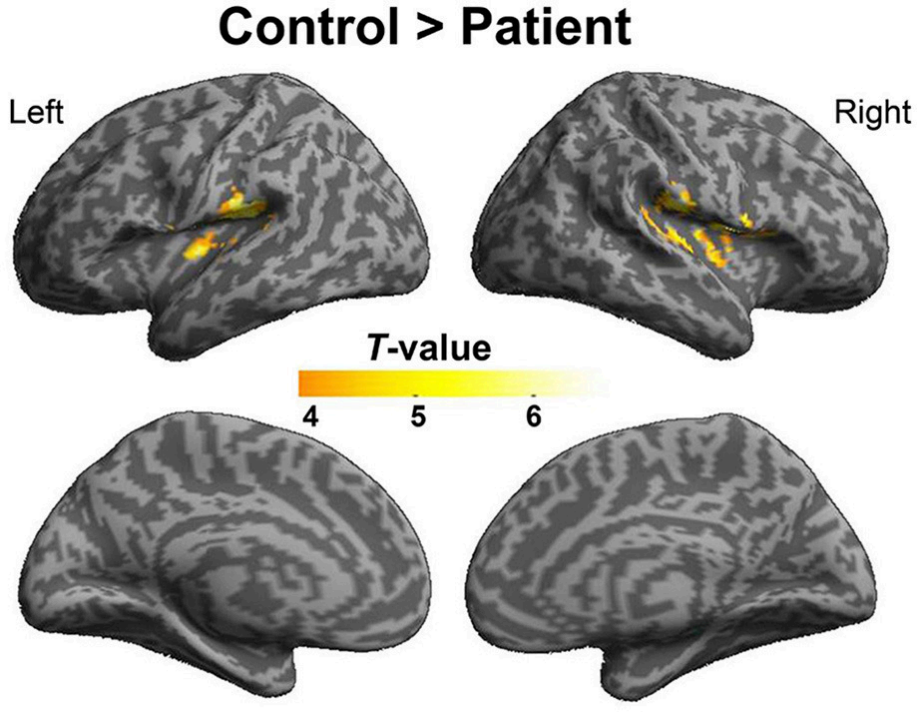
**Difference in brain regions exhibiting differential psychophysical interaction with left posterior inferior temporal gyrus associated with “VSP vs. VNSP” between healthy participants and people with schizophrenia**. The activation maps were thresholded at *p* < 0.05 (voxel-wise FDR corrected, *T* > 4.86) and overlaid on a template brain surface of inflated cortex from SPM8. VSP, visual speech priming; VNSP, visual non-speech priming.

To explore the difference in functional connectivity with the left pITG associated with the “VSP > VNSP” contrast between healthy participants and participants with schizophrenia statistically, an independent two-sample *t*-test was conducted. Compared with healthy participants, reduced functional connectivity with left pITG induced by VSP was found in the bilateral rolandic operculum, bilateral STG, and left insular in participants with schizophrenia. No significantly enhanced functional connectivity was found in participants with schizophrenia relative to healthy participants (Figure 7 and Table 3).

**Table 3:** **Difference in brain regions exhibiting differential connectivity with the left posterior inferior temporal gyrus associated with “VSP vs. VNSP” between patients with schizophrenia and healthy controls**.

**Contrast**	**Coordinates**			**Statistics**			**Location**
	***X***	***Y***	***Z***	***k***	***T***	***Z*-score**	
Control > Patient	−57	−28	18	43	5.72	5.19	L STG
	−42	−28	14	87	5.63	5.12	L RO
	−36	−13	2	56	4.88	4.53	L Insular
	60	−19	18	260	5.68	5.15	R RO
	54	−25	2	41	5.58	5.08	R STG
Control < Patient^a^							

*All peaks and clusters are significant at p < 0.05 (voxel-wise FDR corrected with an extent threshold of more than 40 voxels; T > 4.86). MNI coordinates, k (number of voxels), T-value, and Z-scores are provided. RO, Rolandic operculum; STG, superior temporal gyrus; L, left; R, right*.

a *No significant voxels were obtained at the threshold of p < 0.05 with voxel-wise FDR correction*.

## Discussion

This study, for the first time, investigated the brain substrates underlying the unmasking effect of VSP on target speech recognition in healthy people, and the mechanisms underlying the impaired unmasking effect of VSP in people with schizophrenia. The results suggest that the left pITG may play a critical role in mediating the unmasking effect of VSP in healthy people, and the behavioral reduction in the VSP effect in people with schizophrenia may be related to degraded activation and functional connectivity of the left pITG.

### Brain regions related to the unmasking effect of VSP

The results of behavioral testing in this study confirm previous reports that in a “cocktail-party” listening environment, compared to healthy listeners, listeners with schizophrenia show reduced ability in using temporally pre-presenting VSP cues to improve target-speech recognition against speech masking (Wu et al., 2013a,b).

One of the important findings of this study is that among the 4 VSP-activated brain regions (left pITG, right pITG, left FG, right FG), only the VSP-induced activation of the left pITG is significantly correlated with the VSP-induced (behavioral) improvement of target-speech recognition across healthy listeners. The left pITG is therefore recognized as the “unmasking-correlated brain region.”

Four essential neural mechanisms may simultaneously underlie the unmasking effect of VSP: (1) the brain substrates for processing speech information contained in lipreading, (2) the working memory system retaining VSP signals throughout the co-presentation of the target speech and the masking speech, (3) the central cross-modal integration between visual-speech (lipreading) signals and auditory-speech signals (including the perceptual matching between the phonological/semantic signals of visual lipreading and those of auditory target speech), and (4) the brain substrates for selective attention on target speech and suppression of irrelevant masking signals.

Evidence suggests that the ITG may be the only one brain region that is involved in all the four mechanisms essential to the unmasking effect of VSP: (1) Visual lipreading efficiently activates the ITG (Ludman et al., 2000; Campbell et al., 2001; Xu et al., 2009). For example, the Ludman et al. study (2000) has shown that against a baseline condition where people passively view a static image of a talker's face, lipreading of the talker's speech activates the ITG. (2) There is evidence showing that the ITG plays a critical role in mediating visual working memory (Ranganath et al., 2004; Ranganath, 2006; Woloszyn and Sheinberg, 2009). (3) The left pITG is one of the multi-modal semantic-processing areas associated with the meaning of speech (Wise et al., 1991; Vandenberghe et al., 1996; Mummery et al., 1999; Giraud and Truy, 2002). (4) The ITG is involved in selective attention to attended signals and suppression of distractive signals (Chelazzi et al., 1993, 1998; Zhang et al., 2011).

The absence of correlation between VSP-induced activation of the right ITG and the VSP-induced behavioral improvement may suggest that the right ITG is functionally different from the left ITG in the VSP-induced unmasking process. It has been reported that both the left and right ITG can be activated by observation of non-speech face gestures (Bernstein et al., 2011), speaking faces (Ludman et al., 2000; Campbell et al., 2001), or symbolic gestures (Xu et al., 2009). The left and right ITG are also involved in working memory for visual object (Ranganath et al., 2004; Ranganath, 2006; Woloszyn and Sheinberg, 2009) and visual attentional processes (Chelazzi et al., 1993, 1998; Zhang et al., 2011). However, the left ITG, but not the right ITG, is activated by the processing of brief speech sounds (Alain et al., 2005), discrimination of speech sounds (Ikeda et al., 2010), resolution of semantic ambiguity in spoken sentences (Rodd et al., 2012), integration of auditory and visual signals (Romanski, 2012), or comprehension of speech signals (Giraud and Truy, 2002). Thus, although the right ITG shares some functions with the left ITG, it does not seem to be involved in more specific, more complex, and higher-order processing of speech signals.

In this study, the bilateral FG were also activated by the VSP-listening condition compared to the VNPS condition. It has been reported that the bilateral FG are involved in the processing of “face-like” features of visual objects (Rangarajan et al., 2014) and neurons in the left FG are involved in word recognition (Thesen et al., 2012). The activation of bilateral FG under the VSP-listening condition suggest an involvement of the FG in the early-stage processing of dynamic face signals during speech lipreading.

### VSP-enhanced activation of the ITG is lower in people with schizophrenia

It has been reported that relative to healthy people, people with schizophrenia show less activation in the left pITG while performing the silent lip-reading task (Surguladze et al., 2001). In this study, compared to that in healthy participants, activation of the left pITG induced by VSP was significantly reduced in people with schizophrenia, suggesting a schizophrenia-related functional damage to the left pITG. Clearly, the impaired functions of the left pITG, such as those of general encoding of visual symbolic gestures, visual working memory, multi-modal semantic processing, and visual selective attention, are all important issues in the investigation of schizophrenia.

In this study, in addition to those in the left pITG, reduced BOLD signals were also found in the bilateral TriIFG, left postcentral, left STS, left caudate, left fusiform, right precentral, and right thalamus. The results suggest that under speech masking conditions with visual priming, the impaired target-speech perception in people with schizophrenia may be related to lower activation in these brain areas that are involved in processing masked speech (Scott and McGettigan, 2013), speech production (Scott and McGettigan, 2013; Ding et al., 2016), semantic processing (Huth et al., 2016), or general face-feature processing (Rangarajan et al., 2014).

### Functional connectivity of the left pITG induced by VSP in healthy people

Psychophysiological interaction analyses conducted in this study showed that in healthy participants, the VSP-induced enhancement of functional connectivity occurred from the left pITG to a variety of brain structures, including the temporal areas (bilateral STG), frontal areas (bilateral mSFG, righ MFG, and right IFG), sensor-motor cortices (SMA and supramarginal area), and insular.

The STG is an early stage in the cortical network for speech identification and perception (Hickok and Poeppel, 2004; Ahveninen et al., 2006; Scott and McGettigan, 2013). Both brain-imaging studies (Friederici et al., 2003; Ahveninen et al., 2006) and functional-lesion studies (Boatman, 2004) have shown that the STG is involved in speech perception at the phonetic, lexical-semantic, and syntactic levels. Unmasking-correlated functional connectivity between the left pITG and the left STG observed in this study suggests that the unmasking effect of VSP may be based on the integration between the visual-speech processing and the auditory-speech processing.

The right IFG is involved in both detection of speech stimuli (Vouloumanos et al., 2001) and speech-production process such as lexical decision (Carreiras et al., 2007) and production of lexical tones (Liu et al., 2006). The enhanced functional connectivity of the ITG with the right IFG suggests an enhanced involvement of both the speech-detection system and the speech-production system to deal with “cocktail-party” speech-listening situations.

The mSFG is involved in both controlling goal-directed behavior through the stable maintenance of task sets (Dosenbach et al., 2007) and selecting action sets (Rushworth et al., 2004). Previous studies have also suggested that the SMA may play a role in planning, preparing, controlling, and executing complex movements (Nachev et al., 2008; Price, 2012). The MFG is involved in suppressing irrelevant distracters to ensure accurate target selection in the competition between target and distracters (Lesh et al., 2011; Sokol-Hessner et al., 2012; Jeurissen et al., 2014; Zheng et al., 2016). The insular cortex is implicated in response inhibition (Menon et al., 2001).

Thus, introducing the VSP listening condition may not only induce a mechanism specifically underlying the unmasking effect of VSP, but also generally enhance cooperation of brain areas related to attentional selection of target lipreading signals, suppression of masking signals, visual-auditory speech integration, and facilitation of the functional integration between the earlier-stage visual processing system and the motor executing system.

### Altered functional connectivity of the left pITG in people with schizophrenia

In this study, compared to healthy participants, participants with schizophrenia showed reduced functional connectivity of the left pITG with the bilateral rolandic operculum, bilateral STG, and the left insular.

It is known that the left rolandic operculum (which is caudally adjacent to Broca's area) is involved in both sentence-level speech prosody processing (Ischebeck et al., 2008) and syntactic encoding during speech production (Indefrey et al., 2001). The reduced functional connectivity of the left pTIG with the left rolandic operculum may be related to the schizophrenia-induced impairment of the unmasking effect of VSP.

In addition, reduced functional connectivity of left pITG with the bilateral STG (for the contrast of VSP vs. VNSP) may imply an abnormal integration between the processing of VSP signals and that of auditory speech signals. Moreover, the reduced functional connectivity of the left pITG with the insular may be related to a schizophrenia-induced reduction of inhibition of masker signals or schizophrenia-induced abnormality of emotional processes (also see Menon et al., 2001).

## Conclusions

1. The unmasking effect of VSP on speech recognition against speech masking may be normally associated with both enhanced activation of the left pITG and facilitated integration of the functional network centered at the left pITG.
2. The facilitated integration of the functional network centered at the left pITG may improve both the processing of target-speech signals and the suppression of masker signals.
3. Both VSP-induced activation of the left pITG and functional connectivity of the left pITG with the brain regions related to either speech processing (e.g., bilateral temporal cortex and rolandic Operculum) or inhibition of irrelevant signals (insular) markedly decline in people with schizophrenia, who exhibit impairment in the unmasking effect of VSP on speech recognition.
4. The impairment of the unmasking effect of VSP in people with schizophrenia may be associated with the functional deficits of the brain network centered at the left pITG.
5. Future studies will add other multisensory integration tasks to the protocol described in this study to explore the brain network whose functional deficits are more specific to schizophrenia.

## Author contributions

CW: Experimental design, experiment set up, experiment conduction, data analyses, figure/table construction, paper writing. YZ: Experimental design, experiment set up, data collecting, data analyses, paper writing. JL: Experimental design, experiment set up, experiment conduction, data collecting, and paper writing. BZ: experiment conduction, data analyses. RL: Experiment conduction and data collection. HW: Experiment conduction and data collection. SS: Experiment conduction and data collection. SL: Experiment conduction and data collection. HP: Experiment conduction and data collection. YN: Experimental design, paper writing. LL: Experimental design, figure/table construction, paper writing.

### Conflict of interest statement

The authors declare that the research was conducted in the absence of any commercial or financial relationships that could be construed as a potential conflict of interest.
