# Supplementary material for: Activation and Functional Connectivity of the Left Inferior Temporal Gyrus during Visual Speech Priming in Healthy Listeners and Listeners with Schizophrenia
Source: Front Neurosci. 2017 Mar 15;11:107. doi: 10.3389/fnins.2017.00107 (PMC5350153; doi:10.3389/fnins.2017.00107)
Supplement: Supplementary file 1 [file Presentation1.pdf]

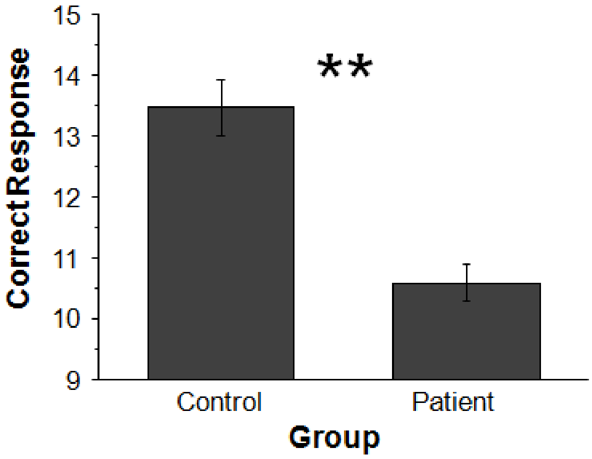

Figure S1. Difference in percentage of correct button-press response for recognizing target speech against speech masking during fMRI scanning between healthy controls and participants with schizophrenia. ** $p < .001$

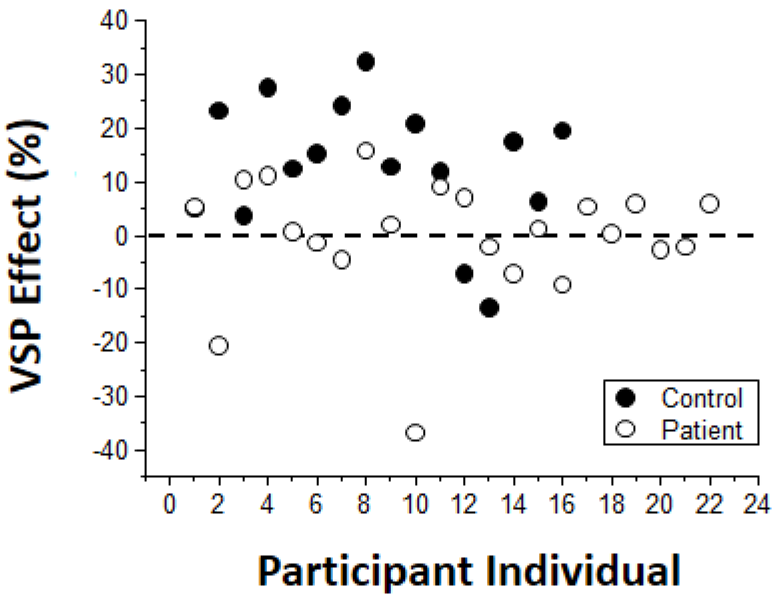

Figure S2. The scatter plot of VSP Effects of healthy controls and people with schizophrenia in the behavioral testing.

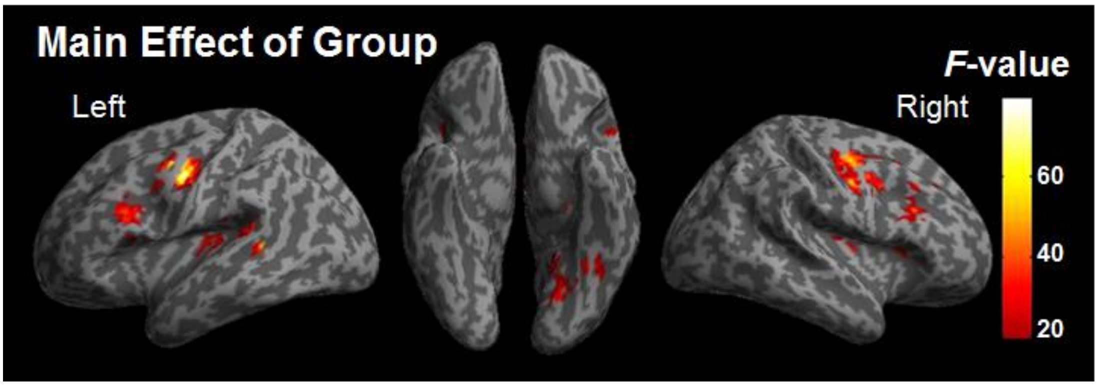

Figure S3. Voxels that exhibited a main effect of group difference (healthy controls versus people with schizophrenia). The activation map is thresholded at $p < 0.05$ with voxel-wise false discovery rate (FDR) correction (F value > 19.51).

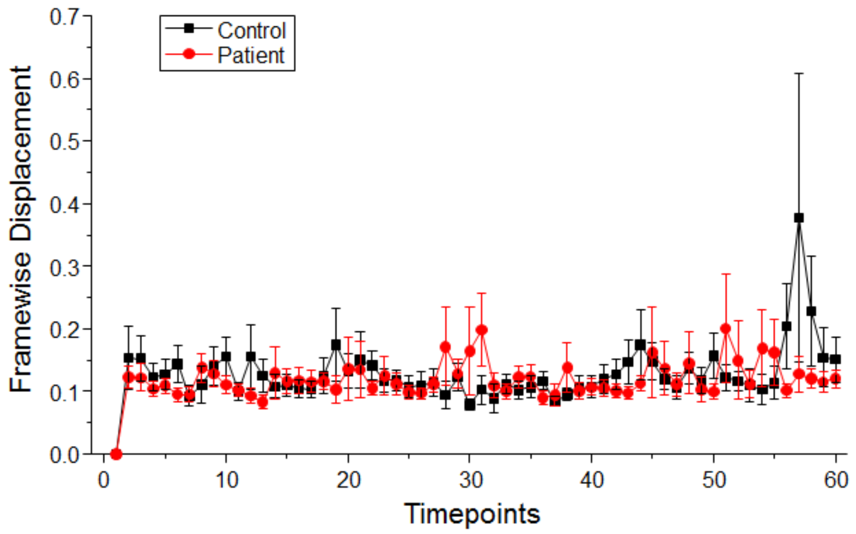

Figure S4. Comparison in framewise displacement (FD) between controls and patients. Error bars indicate the standard errors of the mean.

Table S1. MNI Coordinates of the Brain Regions Revealed by the Group-Level ANOVA of Whole-Brain Data

**Contrast**	**Coordinates**			**Statistics**				**Location**
	X	Y	Z	k	*p* (FDR)	*F*-value	*Z* score	
Main Effect	-45	-10	38	57	.000	59.531	6.461	L Postcentral
(Control > Patient)	42	-7	34	432	.001	43.576	5.701	R Precentral
	-12	-4	14	40	.001	40.358	5.521	L Caudate
	-51	-43	14	60	.001	40.399	5.523	L STS
	39	20	18	95	.005	35.127	5.204	R TriIFG
	-21	-64	-18	93	.022	28.876	4.773	L Fusiform
	27	-28	6	52	.024	28.430	4.740	R Thalamus
	-39	17	22	74	.025	27.951	4.704	L TriIFG
	-36	-52	-18	56	.034	26.071	4.558	L pITG

Note For the 2 (group: control, patients) * 2 (condition: ASP > baseline, ANSP > baseline) ANOVA, only the main effect of group was significant at $p < .05$ (voxel-wise FDR corrected with an extent threshold of more than 40 voxels; F value > 19.51). MNI coordinates, k (number of voxels), T-value, and Z scores are provided. pITG = posterior inferior temporal gyrus; STS = superior temporal sulcus; TriIFG = pars triangularis of inferior frontal gyrus; L = left; R = right.

Table S2. Brain Regions Exhibiting Differential Connectivity with Left Posterior Inferior Temporal Gyrus Associated with "VSP versus VNSP" and "VSNP versus VSP" in Healthy Controls and Participants with Schizophrenia

Group	Contrast	Coordinates			Statistics			Location
		X	Y	Z	k	*T*	*Z*-score	
Control	VSP > VNSP	-33	-52	-34	78	7.19	5.48	L Cerebellum6
		-39	-58	-34	54	5.64	4.64	L Cerebellum_Crus1
		-36	-16	-2	57	6.70	5.23	L Insular
		-57	-13	-10	42	6.48	5.11	L MTG
		39	-25	-2	205	6.51	5.13	R STG
		-39	-40	62	47	6.14	4.93	L Postcentral
		-42	-7	58	43	4.79	4.11	L Precentral
		3	-79	-42	169	6.03	4.87	Cerebelum_7b_R
		36	-55	-38	142	5.25	4.41	Cerebelum_Crus1_R
		-51	8	14	51	5.78	4.72	L OperIFG
		9	47	26	115	5.70	4.68	R mSFG
		3	44	42	77	5.10	4.31	L mSFG
		36	38	42	41	5.62	4.63	R MFG
		-63	-37	26	117	5.03	4.27	L SupraMarginal
		-9	8	46	40	4.46	3.89	LSMA
	VSP < VNSP	42	-43	2	42	6.95	5.35	R STG
		39	-67	6	51	5.28	4.43	R MOC
Patient	VSP > VNSP	-15	17	42	52	5.15	4.52	L SFG

VSP < VNSP	-42	-31	14	108	5.86	5.00	L RO
	36	-16	14	46	5.83	4.98	R Insular
	51	-1	10	108	5.29	4.62	R RO
	-30	-70	-2	63	5.50	4.76	L Fusiform
	21	-19	-2	75	4.88	4.33	R Thalamus
	-12	-37	-2	70	4.61	4.13	L Lingual

All peaks are significant at $p < 0.05$ (voxel-wise FDR corrected with an extent threshold of more than 40 voxels; T value > 4.46 for patient group and T value > 4.21 for control group). MNI coordinates, k (number of voxels), T-value, and Z scores are provided. MTG = middle temporal gyrus; MOC = middle occipital cortex; OperIFG = pars opercularis of inferior frontal gyrus; RO = Rolandic operculum; mSFG = medial superior frontal gyrus; STG = superior temporal gyrus; L = left; R = right.
